# Spexin ameliorated obesity-related metabolic disorders through promoting white adipose browning mediated by JAK2-STAT3 pathway

**DOI:** 10.1186/s12986-024-00790-3

**Published:** 2024-04-24

**Authors:** Bihe Zeng, Qin Shen, Bo Wang, Xuan Tang, Jiaqi Jiang, Yiming Zheng, Hongbiao Huang, Wenyu Zhuo, Wang Wang, Yang Gao, Xuan Li, Shuhui Wang, Wenjie Li, Guanghui Qian, Jie Qin, Miao Hou, Haitao Lv

**Affiliations:** 1 Department of Cardiology, Children’s Hospital of Soochow University, 215025 Suzhou, China; 2 Department of Pediatrics, Affiliated Huai’an Hospital of Xuzhou Medical University, 223002 Huai’an, China

**Keywords:** Spexin, Obesity, Adipose tissue, Browning, JAK2/STAT3 pathway

## Abstract

**Background:**

Spexin, a 14 amino acid peptide, has been reported to regulate obesity and its associated complications. However, little is known about the underlying molecular mechanism. Therefore, this study aimed to investigate the effects of spexin on obesity and explore the detailed molecular mechanisms in vivo and in vitro.

**Methods:**

Male C57BL/6J mice were fed a high-fat diet (HFD) for 12 weeks to induce obesity, and mice fed a standard fat diet were used as controls. Then, these mice were treated with SPX or Vehicle by intraperitoneal injection for an additional 12 weeks, respectively. The metabolic profile, fat-browning specific markers and mitochondrial contents were detected. In vitro, 3T3-L1 cells were used to investigate the molecular mechanisms.

**Results:**

After 12 weeks of treatment, SPX significantly decreased body weight, serum lipid levels, and improved insulin sensitivity in HFD-induced obese mice. Moreover, SPX was found to promote oxygen consumption in HFD mice, and it increased mitochondrial content as well as the expression of brown-specific markers in white adipose tissue (WAT) of HFD mice. These results were consistent with the increase in mitochondrial content and the expression of brown-specific markers in 3T3-L1 mature adipocytes. Of note, the spexin-mediated beneficial pro-browning actions were abolished by the JAK2/STAT3 pathway antagonists in mature 3T3-L1 cells.

**Conclusions:**

These data indicate that spexin ameliorates obesity-induced metabolic disorders by improving WAT browning via activation of the JAK2/STAT3 signaling pathway. Therefore, SPX may serve as a new therapeutic candidate for treating obesity.

## Introduction

Over the last 40 years, the prevalence of obesity has almost tripled worldwide [1], and now obesity is becoming a global public health issue [2]. Obesity increases the risk of many chronic diseases, including type 2 diabetes, cardiovascular diseases, and nonalcoholic fatty liver disease [3]. Obesity is driven by an imbalance between caloric intake and energy expenditure, with excess nutrients stored as body fat [4]. The methods of obesity treatment are based on either reducing calorie intake or increasing energy expenditure. However, reducing the amount of food intake can be difficult in the long run. Thus, enhancing energy expenditure in key metabolic organs, such as adipose tissue, is emerging as an appealing strategy to combat obesity in recent years.

Traditionally, adipose tissues have been divided into two main categories: white adipose tissue (WAT), which stores excess fat [5], and brown adipose tissue (BAT), which functions in thermogenesis by generating heat through the combustion of nutrients that are uncoupled from ATP production by the action of UCP1 [6]. Recent evidence has highlighted the plasticity of WAT, through a range of mechanisms, adipose tissue could switch from energy storing white adipocytes to thermogenic beige adipocytes, and this process is termed ‘browning’ of WAT [7, 8]. These beige cells are closer to the white adipocyte cell lineage and contain comparable amounts of mitochondria as fully stimulated brown adipocytes, suggesting that they may have similar thermogenic capacities [9, 10]. It has been revealed BAT exert beneficial metabolic effects in human [11], and the amount of BAT positively correlates with energy expenditure [12, 13]. Therefore, the induction of adipocyte browning has increasingly been considered to be a target for combating the development of obesity and metabolic syndrome [12, 14].

Spexin (SPX), a novel peptide hormone composed of 14 amino acids, was initially identified using a computational method based on Markov’s model screening of biologically active peptides [15]. This peptide is widely distributed in endocrine and epithelial tissues, including the liver [16], hypothalamus [17], adipose tissue [16], thyroid and anterior pituitary [18] of various species. The potential use of SPX as an anti-obesity treatment has been reported in recent studies. Mona A. Said and colleagues reported that administration of SPX led to a reduction in body weight of mice fed a high-fat diet, suggesting the possibility of using SPX as an anti-obesity treatment [19]. Consistently, Liping et al. reported that exogenous SPX treatment resulted in weight loss in high-fat-diet (HFD)-induced rats [20]. However, the underlying mechanism of SPX in treating obesity remains unclear, particularly regarding its role in promoting white adipose browning.

Therefore, the current study aimed to investigate whether SPX alleviates HFD-induced obesity by promoting white adipose browning and explore the underlying mechanism in vivo and in vitro.

## Materials and methods

### Cell culture, antibodies, chemicals, reagents and antibodies

Mouse 3T3-L1 preadipocytes were obtained from the Procell Life Science and Technology Co. Ltd (China). Antibodies UCP1 (ab10983), TBX1 (ab109313) and CIDEA (ab8402) were purchased from Abcam (Cambridge, UK), Actin (AP0063), JAK2 (3230), STAT3 (12,640), p-JAK2 (3771) and p-STAT3 (9145) were from Cell Signaling Technology (Boston, MA, USA), and JAK2-specific inhibitor AZD1480 (S2162), STAT3-specific inhibitor stattic (S7024) was from Selleck.cn (Shanghai, China).

### Animals

**Fig. 1:**
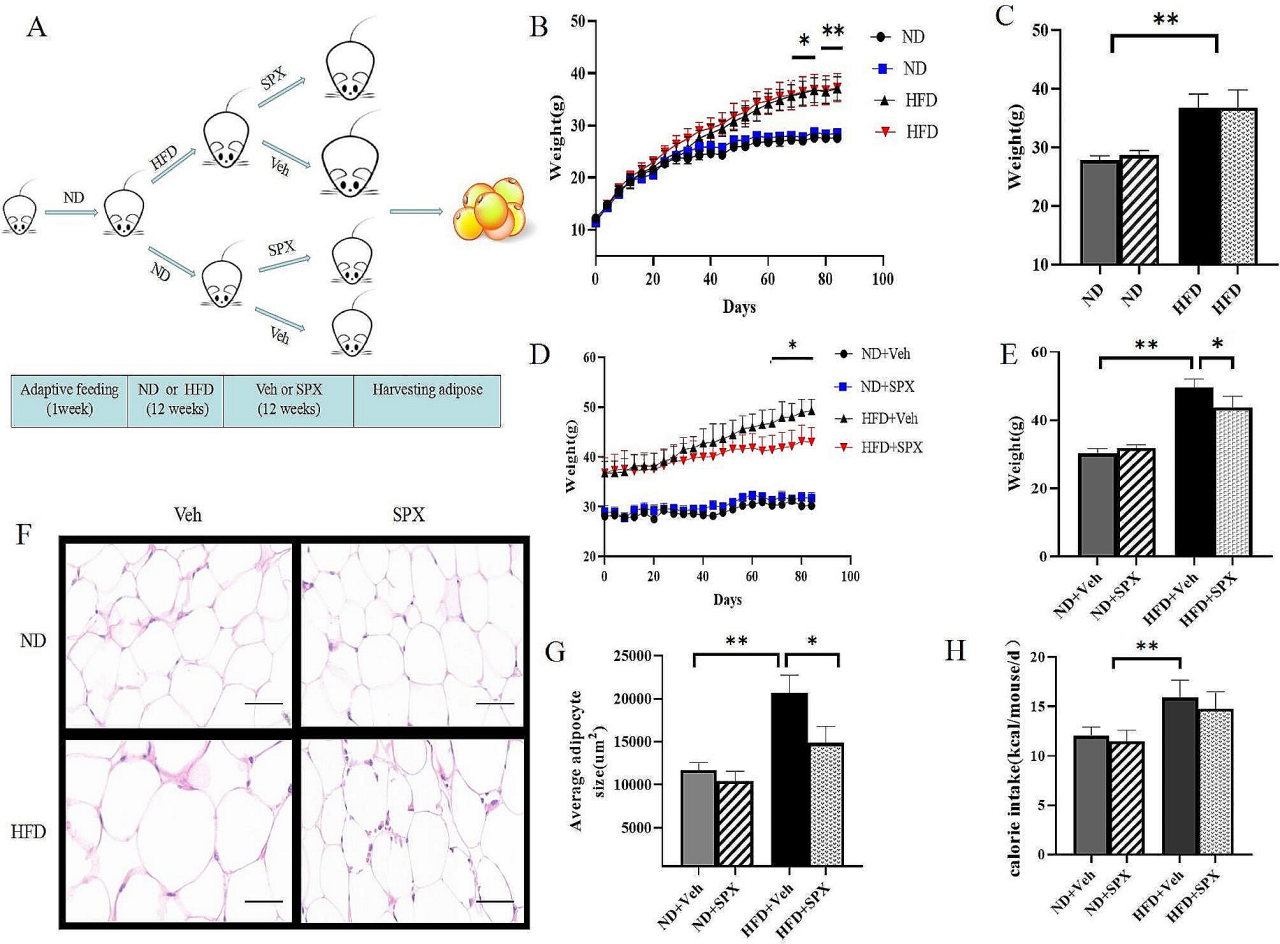
SPX ameliorates high-fat-diet-induced obesity in mice. **(A)** Schematic description of animal experiments. 3-week-old C57BL/6J male mice after one-week adaptive feeding were divided randomly into a high-fat diet group (HFD) and a normal diet (ND) group. After 12 weeks, HFD or ND mice were half received SPX injection (30 µg/kg/day; HFD + SPX or ND + SPX) or vehicle injection (HFD + Veh or ND + Veh). **(B)** The body weight during the protocol. **(C)** Obesity model successfully established after 12 weeks. **(D, E)** Body weight of mice after SPX or Veh treatment at the end of the study. **(F)** H&E staining in WAT (epididymal WAT) (scale bar: 100 μm). **(G)** Adipocyte size in WAT of the mice. **(H)** The food intake after SPX or Veh treatment among each group. All values are represented as means with error bars representing S.D. *, *P* < 0.05; **, *P* < 0.01. *n* = 6 for each group

Forty male C57BL/6J mice (3 weeks of age, obtained from Shanghai Laboratory Animal Center weighing about 10–12 g) were housed in isolated animal cages (5 mice/cage) on standard laboratory conditions (22–24℃ surrounding temperature, 40–60% relative humidity and 12–12 h light-dark cycle) with free access to their experimental diet and water all over the time of the experiment. C57BL/6J male mice were randomly assigned into 4 groups: (1) normal diet + vehicle group (ND + Veh), in which mice were fed a standard chow (70% Carbohydrate, 10% Fat, and 20% Protein, 4.057 kcal/g) from 4 weeks of age to the end of the study, and intraperitoneally injected with Veh for 12 weeks beginning at 12 weeks after ND; (2) ND + spexin group (ND + SPX), in which mice were fed a standard chow for 24 weeks and intraperitoneally injected with recombinant spexin (30 ug/kg/day) for 12 weeks beginning at 12 weeks after ND; (3) high-fat diet + Veh group (HFD + Veh), in which mice were fed an HFD (20% Carbohydrate, 60% Fat, and 20% Protein, 5.243 kcal/g) for 24 weeks and intraperitoneally injected with Veh for 12 weeks beginning at 12 weeks after HFD; (4) HFD + spexin group (HFD + SPX), in which mice were fed an HFD as well as given spexin (30 ug/kg/day) intraperitoneal injection for 12 weeks beginning at 12 weeks after HFD, and the schematic description of animal experiments was shown in Fig. 1A. Mice body weight and food intake per cage were measured weekly. The calorie intake was calculated by energy content per gram of food × food intake. After 12 weeks of SPX or Veh treatment, these mice were anesthetized by pentobarbital, and then epididymal WAT and blood samples were collected from individual mice. All samples were stored at -80℃ until assay.

### Metabolic rate analysis

Before the metabolic rate was monitored, mice were individually caged for 24 h to acclimate to the system.The oxygen consumption (VO_2_), carbon dioxide production (VCO_2_), respiratory exchange ratio (RER = VCO_2_/VO_2_) and energy expenditure (EE) were determined after 12 weeks of SPX or Veh treatment using a Comprehensive Lab Animal Monitoring System (CLAMS)-Oxymax (Columbus Instruments, Columbus, USA).

### Intraperitoneally glucose tolerance tests

Mice were tested for glucose tolerance after 12 weeks of SPX or Veh treatment. For the intraperitoneal glucose tolerance test (IPGTT), mice were fasted overnight and then intraperitoneally injected with glucose solution at a dose of 2.0 g/kg body weight. Blood glucose concentration was measured before and at 30, 60, 90 and 120 min after injections.

### Assessment of the biochemical parameters

Blood samples were collected through the cardiac puncture in mice, left for 30 min at room temperature toclot and then centrifuged at 3000 rpm for 15 min and the serum was separated and stored at -80℃ for estimation of biochemical parameters.

The levels of triglyceride (TG), total cholesterol (TCHO), high-density lipoprotein cholesterol (HDL) and low-density lipoprotein cholesterol (LDL) were measured with the enzyme method following the manufacturer’s instruction (Roche cobas8000).

### Hematoxylin and eosin (H&E) staining

Fresh adipose tissues were harvested, fixed in 4% paraformaldehyde in PBS (pH 7.4) and processed to form paraffin blocks. Adipocyte size was measured in hematoxylin and eosin (H&E) stained slides using cell Sens software (Olympus, Tokyo, Japan).

### Cell culture

Mouse 3T3-L1 preadipocytes were maintained in DMEM containing 10% FBS and 1% penicillin/streptomycin (P/S) at 37 °C in a 5% CO2 incubator. Sufficiently confluent cells were maintained in a differentiation induction medium consisting of 10 µg/mL of insulin (Sigma), 0.25 µM dexamethasone (Sigma), and 0.5 mM 3-isobutyl-1-methylxanthine (Sigma) in DMEM containing 10% FBS, followed by culture in maturation medium consisting of 10% FBS and 10 µg/ml of insulin in DMEM. The maturation medium was changed every 2 days. By day 10 of differentiation, the mature adipocytes were treated with SPX (100nM), in the presence or absence of pretreated JAK2 inhibitor AZD1480 (2.5 μm) or STAT3 inhibitor stattic (2.5 μm) for 3 h, respectively, then the cells were collected for RNA and protein isolation. All in vitro experiments were repeated at least in triplicate.

### Real-time quantitative PCR (RT-qPCR) analysis

Total RNA was isolated from cells and tissues using TRIzol Reagent (Invitrogen, United States) and then treated with RNase-free DNase and reverse-transcribed to cDNA using 5 × all-in-one RT MasterMix (Takara) according to the manufacturer’s protocol. 2× SYBR Green qPCR MasterMix (Bimake, United States) was employed to quantitatively determine transcription levels of genes with RT-qPCR (Roche Lightcycler® 96, Mannheim, Germany). Sample qPCR assays were run in duplicate, and transcription levels of all genes were normalized to the level of Actin. The sequences of the primer sets used in this study are listed in Table 1.

**Table 1:** List of primers used for real-time quantitative PCR

Gene	Primer sequence
UCP1 - F	CAAAAACAGAAGGATTGCCGAAA
UCP1 - R	TCTTGGACTGAGTCGTAGAGG
CIDEA - F	TGCTCTTCTGTATCGCCCAGT
CIDEA - R	GCCGTGTTAAGGAATCTGCTG
TBX1 - F	CTGTGGGACGAGTTCAATCAG
TBX1 - R	TTGTCATCTACGGGCACAAAG
Actin - F	TGTCCACCTTCCAGCAGATGT
Actin - R	GCTCAGTAACAGTCCGCCTAGA
Cytochrome b - F	CCACTTCATCTTACCATTTATTATCGC
Cytochrome b - R	TTTTATCTGCATCTGAGTTTAATCCTGT
β-actin - F	TGCCGACAGGATGCAGAAG
β-actin - R	TTCCAGCAGATGTGGATCAGC

### Total protein extraction and western blotting

Whole-cell lysates and tissue lysates were washed twice with cold PBS (Procell, China) and then re-suspended in appropriate RIPA lysis buffer (Beyotime, China).

Protein concentration was detected by Enhanced BCA Protein Assay Kit (Beyotime, China). Western blotting was done by electrophoresing 20 µg proteins on SDS–PAGE and subsequently transferring electrophoretically onto PVDF membranes (Millipore, Burlington, MA, USA). Blocking was done in 5% non-fat dry milk in 1×TBST for 2 h at room temperature and then incubated with appropriate primary antibodies at 4 °C overnight. Then the membranes were incubated with secondary antibodies(Jackson, Lancaster, PA, USA) for 1 h at room temperature. The bands were detected with an AmerSham Imager 600 (GE, Boston, MA, USA) and analyzed using ImageJ analysis software. Actin was used as the internal control. Because the molecular weights of target proteins are very close/overlapping, then membranes were cut horizontally.

### Analysis of mitochondrial DNA content

The amount of mitochondrial DNA (mtDNA), extracted and purified using DNAeasy Blood and Tissue Kit (Qiagen), was determined by real-time quantitative PCR of the mitochondrial cytochrome B (CYTB) gene normalized to the nuclear Actingene. The real-time PCR was performed with 100 ng of total DNA using RT-PCR (Roche Lightcycler® 96, Mannheim, Germany) according to the manufacturer’s instructions. The sequences of the primer sets used in this study are listed in Table 1.

### Immunocytochemistry of UCP1

Differentiated mature adipocytes were fixed in 10% formalin for 20 min and permeabilized with 5% bovine serum albumin containing 0.1% Triton X-100 for 2 h. Cells were incubated overnight at 4°C with rabbit polyclonal anti-UCP1 antibody (ab10983, Abcam) (1:500) followed by incubation with secondary Alexa Fluor 488- conjugated donkey anti-rabbit IgG for 1 h. After washing three times, cells were counterstained with 4’, 6- diamidino-2-phenylindole (DAPI) for 15 min. Photographs were taken using an Olympus micro FV10 viewer.

### Mitochondrial contents

The mitochondrial content in differentiated mature 3T3-L1 adipocytes was evaluated using MitoTracker® Green FM (Meilunstar). Cells were stained with 200 nmol/L MitoTracker® Green FM for 45 min in an atmosphere of 37 °C and 5% CO2, followed by one wash with 1×phosphate-buffered saline (1× PBS). The cells were fixed with Hoechst33342 in PBS for 15 min at 37 °C. After washing with 1× PBS, cells were observed by fluorescent microscopy (Olympus).

### Electron microscopic observation

Adipose tissues and differentiated 3T3-L1 adipocytes were washed with 1× PBS and fixed in 2.5% (w/v) glutaraldehyde solution overnight at 4 °C. After washing three times with 0.1 M phosphate buffer (pH 7.2) and fixed in 1% osmic acid at 4℃ for 2 h. The samples were embedded in Epon-Araldite resin for penetration and placed in a model for polymerization. Followed by the counterstaining of 3% uranyl acetate and 2.7% lead citrate. Then observed with an HT7800 transmission electron microscope.

### Statistical analysis

All values are expressed as means ± standard deviation (SD). Statistical analyses were performed using Prism Statistical Package of Social Science (GraphPad Prism 8.0). Data were analyzed using a two-way ANOVA and significant interactions were followed up with Tukey post hoc analysis. Comparisons between the two groups were performed by unpaired Student’s t-tests. The energy metabolism data, including VO2, VCO2 and EE were analyzed by Analysis of Covariance (ANCOVA), with body weight as a covariate [21, 22]. *P* < 0.05 was considered statistically significant.

## Results

### SPX ameliorates high-fat-diet-induced weight gain in mice

As demonstrated in Fig. 1B and C, the HFD led to significant increases in body weight compared with ND. SPX treatment prevented further body weight gain in HFD mice compared with those treated with Veh (Fig. 1D and E). Interestingly, SPX did not reduce the food intake of these HFD mice (Fig. 1H). In addition, there were no differences in the body weight between ND + SPX and ND + Veh mice throughout this study. In line with body weight, H&E staining revealed the mean adipocyte sizes in HFD + SPX mice were smaller than HFD + Veh mice. However, there was no difference in adipocyte sizes between ND + SPX and ND + Veh mice (Fig. 1F and G).

### SPX promotes energy expenditure in HFD mice

**Fig. 2:**
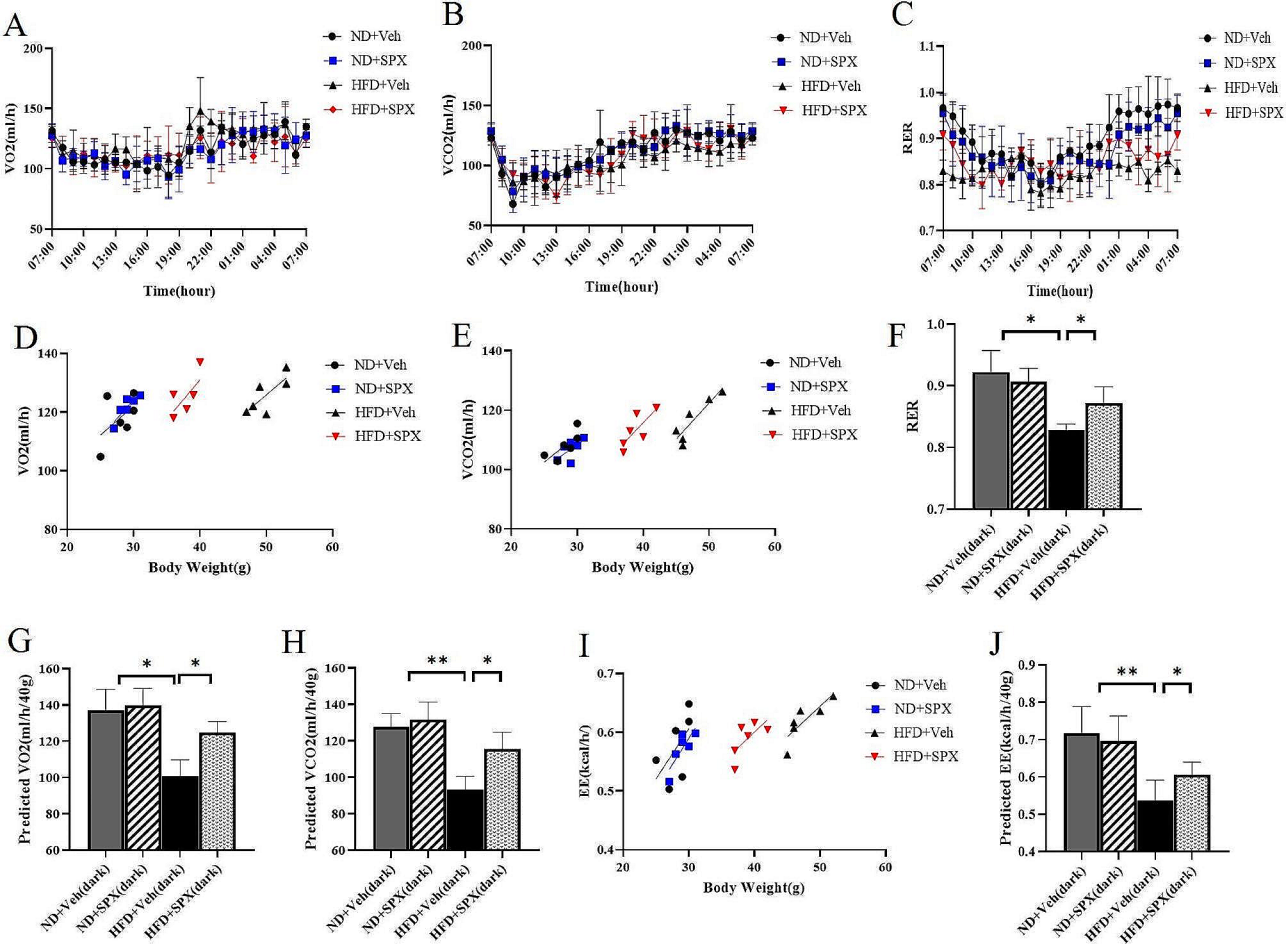
SPX promotes mice energy expenditure. **(A)** The oxygen consumption (VO_2_) of the mice during a 12 h light/12 h dark cycle. **(D)** the average values of VO_2_ for 12 h dark periods against body weight, **(G)** ANCOVA predicted VO_2_ at a given body weight of 40 g. **(B)** The carbon dioxide production (VCO_2_) of the mice during a 12 h light/12 h dark cycle. **(E)** the average values of VCO_2_ for 12 h dark periods against body weight, **(H)** ANCOVA predicted VCO_2_ at a given body weight of 40g. **(C)** the average values of the respiratory exchange ratio (RER) of the mice during a 12 h light/12 h dark cycle. **(F)** the average values of RER for 12-h light/12-h dark periods. **(I)** the average values of energy expenditure (EE) for 12 h dark periods against body weight. **(J)** ANCOVA predicted EE at a given body weight of 40 g. All values are presented as means with error bars representing S.D. In the bar graph, *, *P* < 0.05; **, *P* < 0.01. *n* = 6 for each group

The effect of SPX on energy expenditure was analyzed using a comprehensive laboratory animal-monitoring system. The mice fed with HFD had lower VO_2,_ VCO_2_ and RER levels than the mice fed with ND. After SPX treatment, VO_2_ (Fig. 2A, D and 2G) and VCO_2_ (Fig. 2B, E and 2 H) levels of HFD + SPX mice were higher than HFD + Veh mice. Moreover, SPX treatment significantly increased RER (Fig. 2C and F) and EE (Fig. 2I and J) in HFD + SPX mice compared to HFD + Veh mice. However, no differences in VO_2_, VCO_2_, RER and EE were observed between ND + SPX and ND + Veh mice.

### SPX improves glucose and lipid metabolism in HFD-induced obesity

**Fig. 3:**
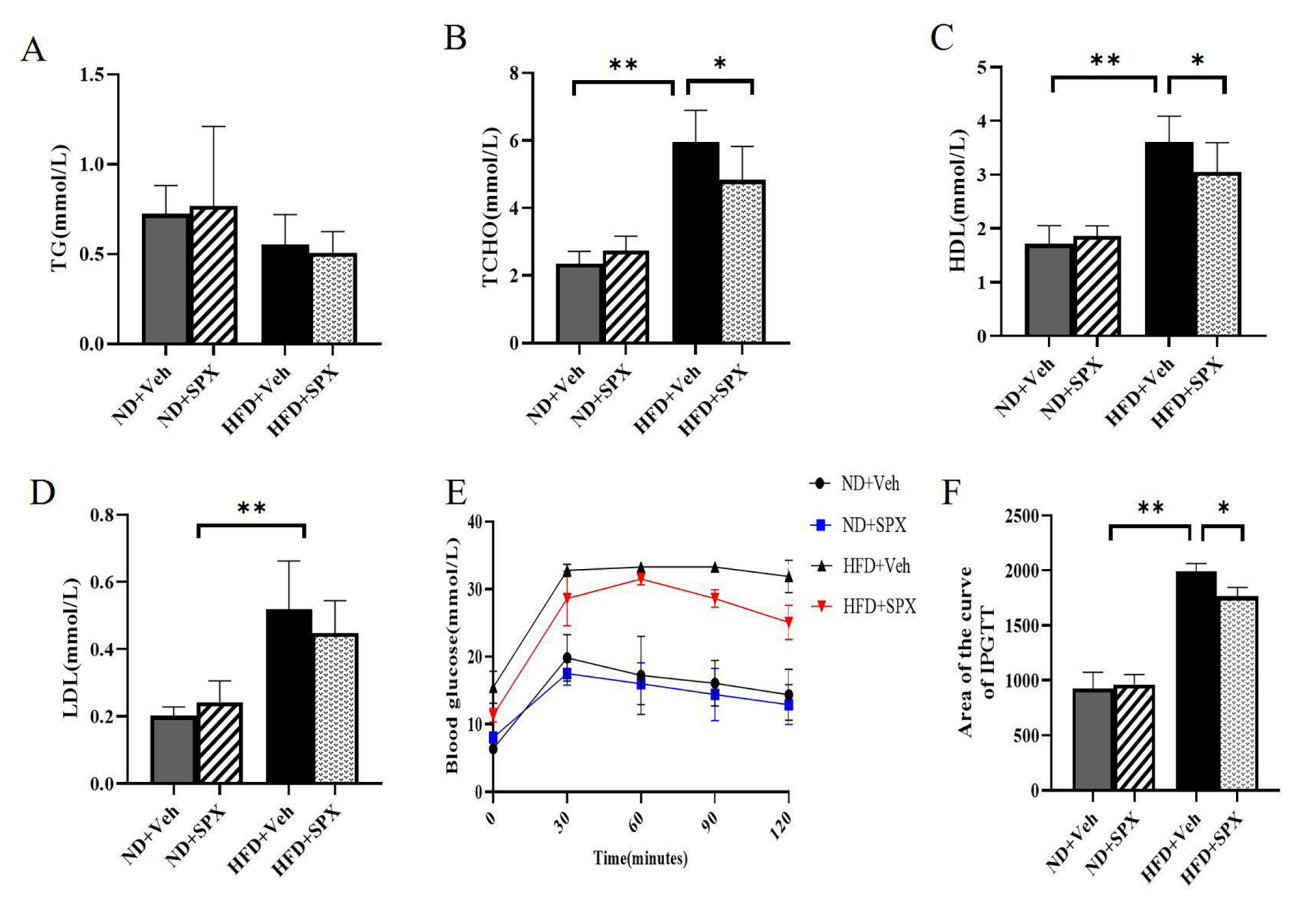
SPX improves lipid metabolism and glucose tolerance in HFD feeding-induced obesity. (**A** ~ **D**) Serum TG, TCHO, HDL and LDL levels of the mice. **(E)** Glucose levels during intraperitoneal glucose tolerance test (IPGTT). **(F)** Glucose area of the curve (AOC) during IPGTT. All values are represented as means with error bars representing S.D. *, *P* < 0.05; **, *P* < 0.01. *n* = 6 for each group

The serum TCHO, LDL and HDL levels were significantly elevated in mice fed HFD compared with the mice fed ND. After SPX treatment, serum TCHO and HDL levels were decreased in HFD + SPX mice compared with HFD + Veh mice. However, there was no significant difference in blood lipid levels between ND + Veh and ND + SPX mice (Fig. 3B and C), and the serum TG levels were comparable among each group (Fig. 3A).

As shown in Fig. 3E, the mice fed with HFD has higher blood glucose levels and larger AOC during IPGTT than the mice fed with ND (Fig. 3E and F), which indicated that HFD impaired glucose tolerance in these mice. Interestingly, the AOC value was significantly reduced in HFD + SPX mice compared with HFD + Veh mice, while there was no difference in AOC between ND + SPX and ND + Veh mice (Fig. 3F). These results suggested that SPX alleviated glucose tolerance in HFD-induced obese mice.

### SPX induces browning and increased mitochondrial number in WAT of HFD mice

**Fig. 4:**
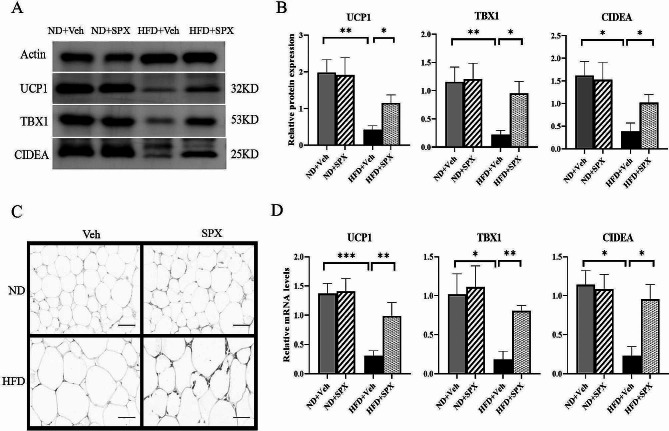
SPX induces thermogenic gene expression and browning in WAT of HFD mice. **(A)** Immunoblotting for indicated proteins in WAT of the mice. The protein **(B)** and mRNA **(D)** expression levels of UCP-1, TBX1 and CIDEA in WAT. **(C)** Immunohistochemical staining of UCP-1 in WAT (scale bar: 100 μm). All values are represented as means with error bars representing S.D. *, *P* < 0.05; **, *P* < 0.01. *n* = 6 for each group

The mRNA and protein expression of UCP1 and browning adipocyte markers, including TBX1 and CIDEA were significantly reduced in HFD mice compared to ND mice. After SPX treatment, the protein (Fig. 4A and B) and mRNA (Fig. 4D) expression of UCP1 and other browning adipocyte markers in WAT of HFD mice were higher than those treated with Veh and the upregulation of UCP-1 in HFD + SPX group was also confirmed by immunohistochemical staining (Fig. 4C).

**Fig. 5:**
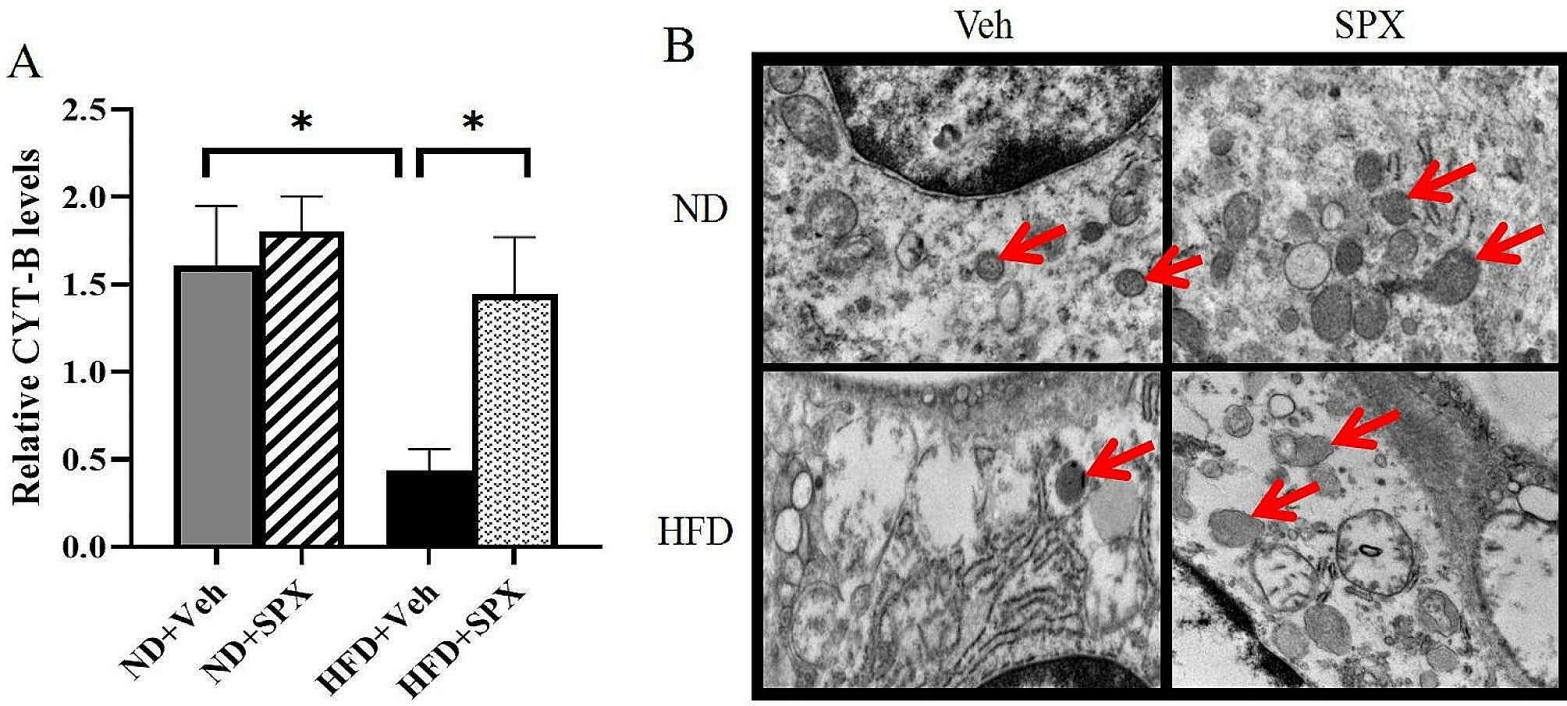
SPX increases mitochondrial biogenesis in WAT of HFD mice. **(A)** The expression levels of mitochondrial biogenesis proteins CYT-B/actin in the mice. **(B)** Representative transmission electron microscopic images (Red arrows indicate the mitochondria). Original magnification, ×15,000. All values are represented as means with error bars representing S.D. *, *P* < 0.05; **, *P* < 0.01. *n* = 6 for each group

We estimated WAT mitochondrial number by measuring mtDNA relative to nuclear DNA (CYT-B/b-actin) and found HF diet significantly reduced the mitochondrial number, while SPX significantly increased the mitochondrial number in WAT of HFD + SPX group compared with HFD + Veh group (Fig. 5A). These data were confirmed by transmission electron microscopy (Fig. 5B). Collectively, SPX drove the browning along with increasing mitochondrial number in WAT.

### SPX increases browning -fat specific markers in 3T3-L1 white adipocytes

**Fig. 6:**
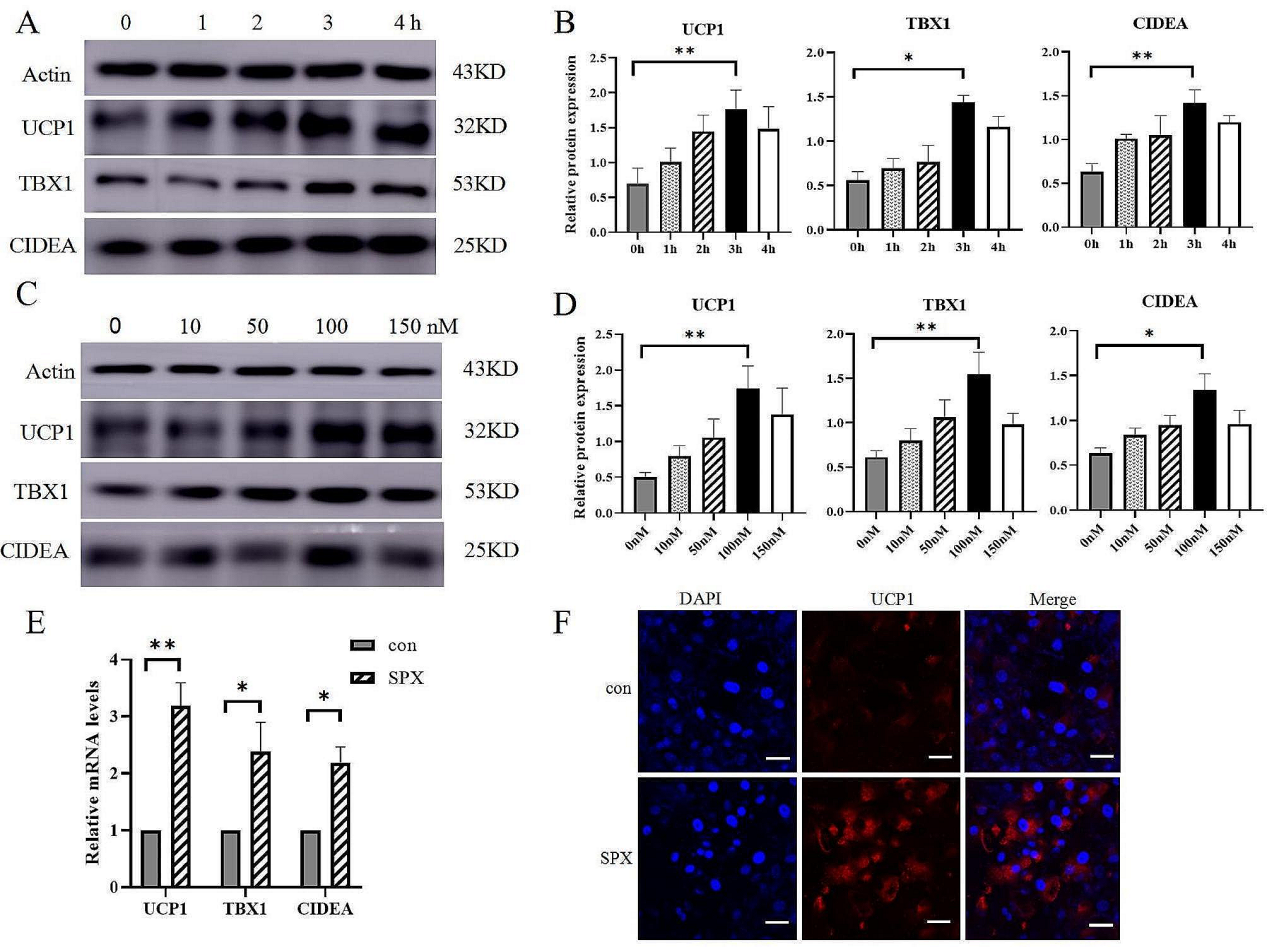
SPX increases beige-fat specific markers in 3T3-L1 white adipocytes. (**A** ~ **D**) SPX increases the expressions of UCP1, TBX1 and CIDEA in 3T3-L1 adipocytes in a time-dependent and concentration-dependent manner. **(E)** The mRNA expression levels of UCP1, TBX1 and CIDEA in 100 nM SPX-treated differentiated 3T3-L1 adipocytes after 3 h. **(F)** Immunofluorescence staining shows increased expression of UCP1 upon 100 nM SPX treatment 3 h (×600 magnification; scale bar = 50 μm). All values are represented as means with error bars representing S.D. *, *P* < 0.05; **, *P* < 0.01. *n* = 6 for each group

To investigate whether SPX also induces white adipocyte browning in vitro, we treated mature 3T3-L1 adipocytes with SPX, and demonstrated that SPX increases the expressions of UCP1, TBX1 and CIDEA in a time-dependent (Fig. 6A and B) and concentration-dependent manner (Fig. 6C and D). Our results showed the optimal concentration was 100nM and the optimal time was 3 h.Therefore, this concentration was selected for further tests. We found the mRNA expression levels of UCP1, TBX1 and CIDEA was significantly upregulated by SPX (Fig. 6E). Immunofluorescence staining also confirmed the upregulation of UCP-1 expression by SPX treatment (Fig. 6F).

### SPX increases mitochondrial number in 3T3-L1 white adipocytes

**Fig. 7:**
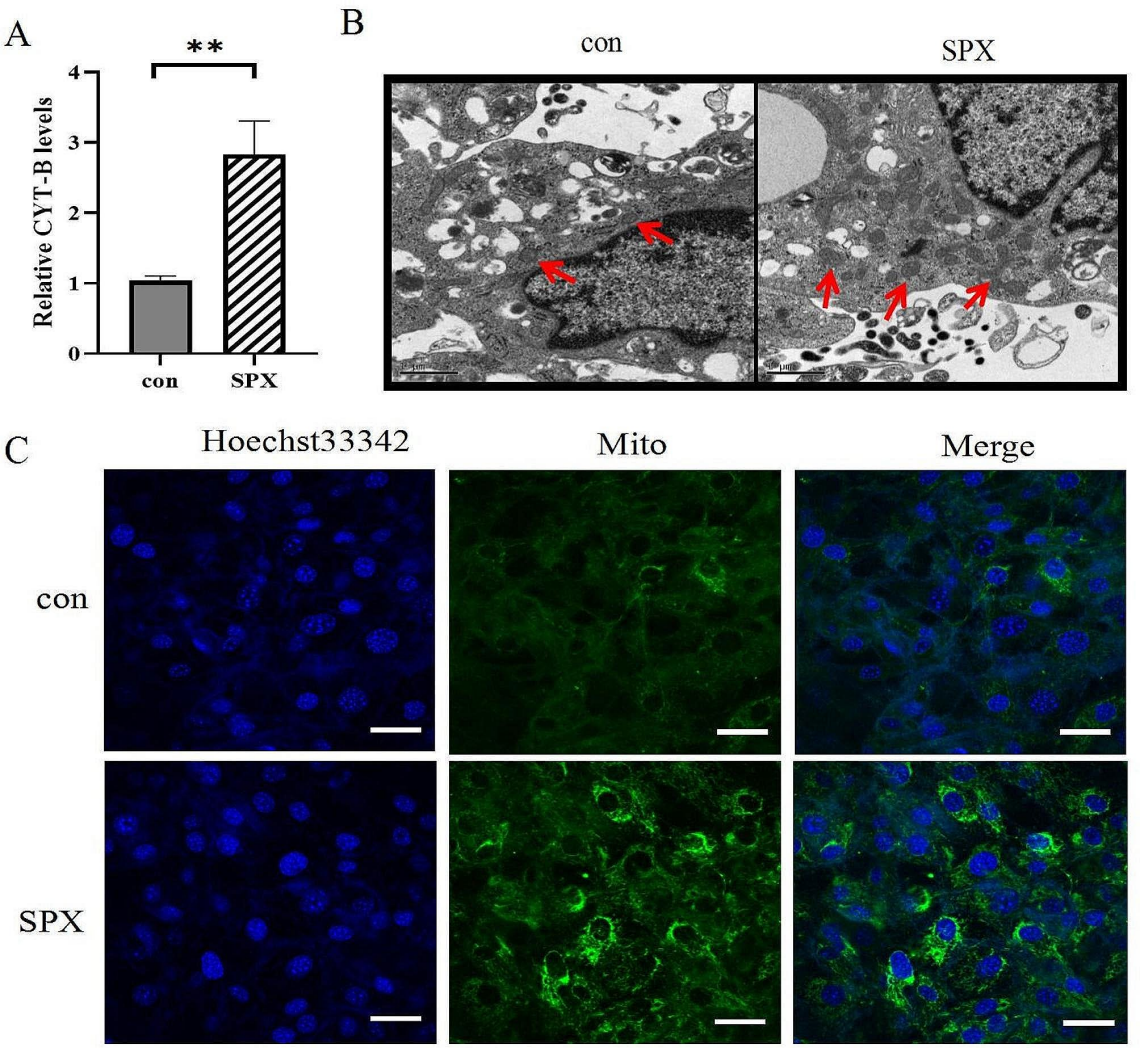
SPX increases mitochondrial biogenesis in 3T3-L1 white adipocytes. **(A)** The levels CYT-B/actinin 100 nM SPX-treated differentiated 3T3-L1 adipocytes after 3 h. **(B)** Representative transmission electronic microscopic images from 100 nM SPX-treated differentiated 3T3-L1 adipocytes. Original magnification, ×15,000 (Red arrows indicate the mitochondria). **(C)** MitoTracker Green staining of mitochondria in 3T3-L1 cells (Original magnification, 600×, scale bar = 50 μm). All values are represented as means with error bars representing S.D. In the bar graph, *, *P* < 0.05; **, *P* < 0.01. *n* = 6 for each group

The CYT-B/Actin levels were higher in SPX-treated adipocytes compared with the control group, which indicates mitochondrial number was significantly upregulated by SPX (Fig. 7A), and the increasing number of mitochondria in SPX-treated adipocytes was also observed by transmission electronic microscopic (Fig. 7B). Accordingly, staining of fully differentiated adipocytes with MitoTracker green revealed stronger staining in SPX-treated adipocytes (Fig. 7C).

### SPX induces browning of white adipocytes via enhancing JAK2-STAT3 signaling pathway

**Fig. 8:**
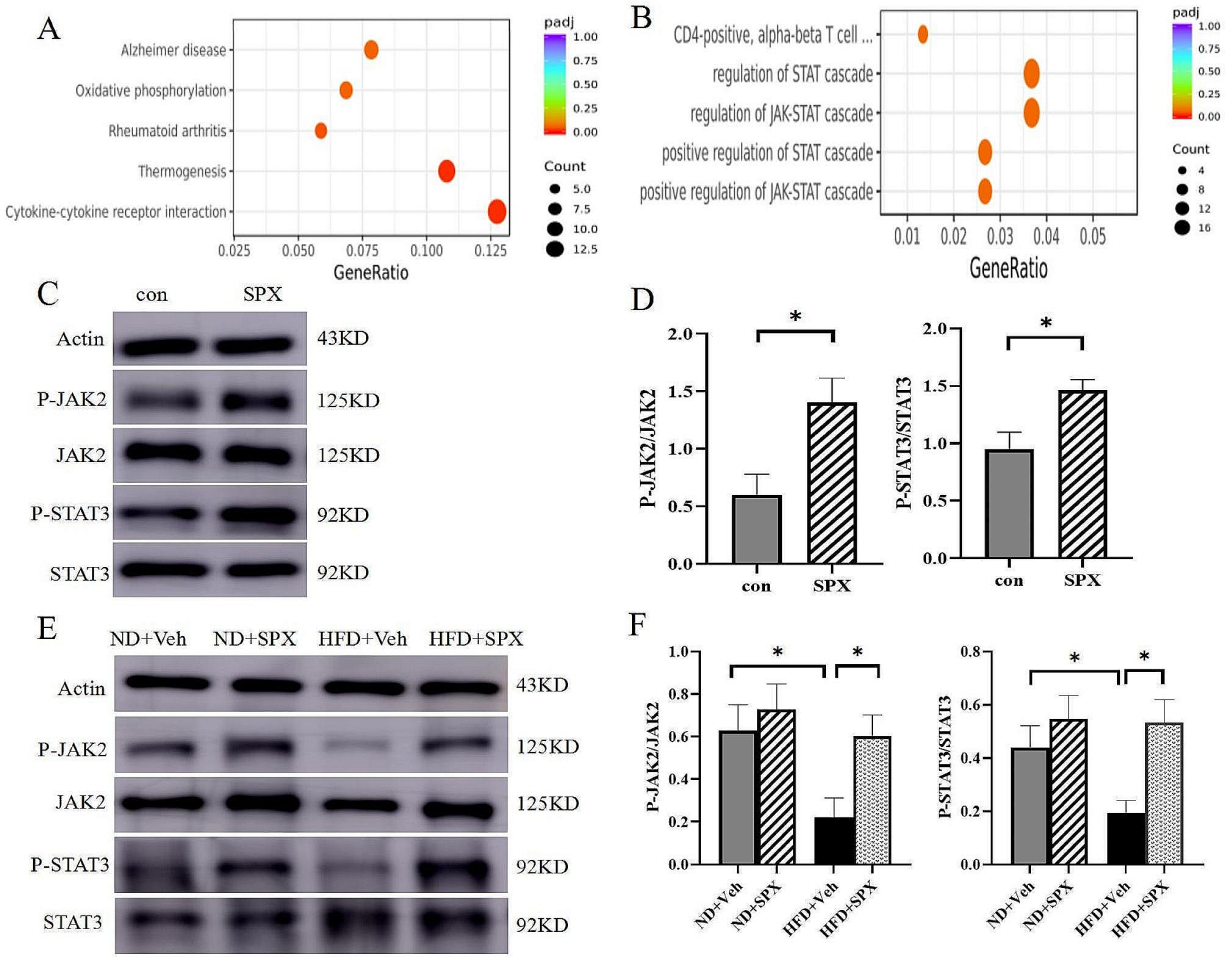
SPX-induced adipose browning is mediated through JAK2 and STAT3 signaling. (**A**, **B**) RNA sequence of the possible involved signaling pathways. (**C**, **D**) The expression of P-JAK2/JAK2 and P-STAT3/STAT3 in 3T3-L1 mature adipocyte. (**E**, **F**) The expression of P-JAK2/JAK2 and P-STAT3/STAT3 in WAT of mice. All values are represented as means with error bars representing S.D. In the bar graph, *, *P* < 0.05; **, *P* < 0.01. *n* = 6 for each group

To investigate the molecular mechanisms underlying SPX regulation of adipocyte browning, RNA-Seq analysis on fully differentiated adipocytes was performed to investigate the global transcriptome changes induced by SPX treatment, the top three enriched terms in the biological process were ‘Cytokine-cytokine receptor interaction’, ‘Thermogenesis’ and ‘Rheumatoid arthritis’ (Fig. 8A), and KEGG enrichment analysis showed that the JAK/STAT signaling pathway was an important pathway involved in the browning action of SPX (Fig. 8B).

**Fig. 9:**
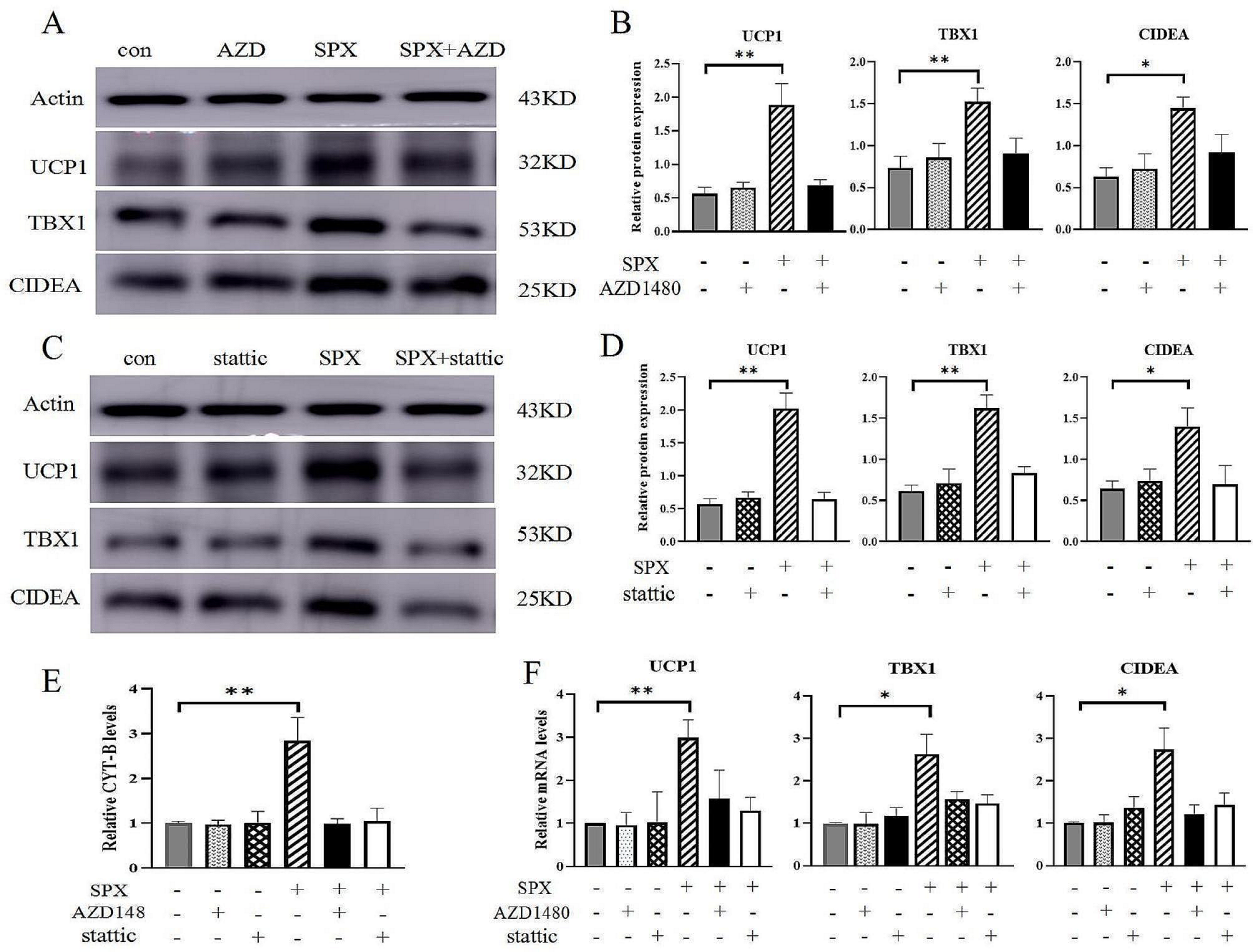
JAK2-STAT3 signaling pathway inhibitors eliminate the adipose browning induced by SPX. The adipocytes were treated with an inhibitor of JAK2 (AZD1480) or an inhibitor of STAT3 (stattic). (**A** ~ **D**) The protein expression of UCP1, TBX1 and CIDEA treated with AZD1480 and stattic. **(E)** The expression levels of mitochondrial biogenesis proteins CYT-B. **(F)** The mRNA expression of UCP1, TBX1 and CIDEA. All values are represented as means with error bars representing S.D. In the bar graph, *, *P* < 0.05; **, *P* < 0.01. *n* = 6 for each group

Consistent with the RNA-Seq result, we demonstrated that expression levels of p-JAK2/JAK2 and p-STAT3/STAT3 were upregulated (Fig. 8C and D). Also, the phosphorylation of JAK2 and STAT3 in WAT of HFD + SPX mice was significantly increased compared to those HFD + Veh mice (Fig. 8E and F). However, there was no difference in JAK2 and STAT3 phosphorylation levels between ND + SPX and ND + Veh groups. Therefore, we sought to explore if SPX promotes the browning of adipocytes via activating the JAK2-STAT3 signaling pathway, and mature adipocytes were pre-treated with JAK2-STAT3 signaling pathway inhibitors, either AZD1480 (JAK2 antagonist) or static (STAT3 antagonist). AZD1480 (Fig. 9A and B) or static (Fig. 9C and D) pretreatment could also eliminate the increase of UCP1, TBX1 and CIDEA. The upregulation of mitochondrial cytochrome B gene induced by SPX in adipocytes was reversed by AZD1480 or static (Fig. 9E). Similarly, AZD1480 or static pretreatment eliminated the increase in UCP1, TBX1 and CIDEA mRNA expression in SPX treated adipocytes (Fig. 9F). These findings indicate that SPX promotes adipocyte browning by activating the JAK2-STAT3 signaling pathway.

## Discussion

Strategies that plasticize WAT to acquire brown-fat like features are a promising approach against obesity and related metabolic complications [23]. In the current study, we demonstrated that SPX alleviated the body weight gain and ameliorated glucose and lipid metabolism in obese mice induced by a high-fat diet, and this effect was due to the pro-browning action in WAT driven by SPX. More importantly, we further identified that its pro-browning was mediated by the JAK2/STAT3 pathway.

SPX is a new peptide with multiple effects, including fatty acid, glucose homeostasis and energy balance. It is reported SPX gene expression was downregulated in the omental and subcutaneous fat tissue of obese subjects [24], and negatively correlated with HOMA-IR and insulin levels in obese women [25]. In the current study, we initially successfully established the mouse obesity model by HFD, then demonstrated that SPX treatment ameliorated weight gain, and improved lipid metabolism as well as glucose tolerance in HFD-fed mice. Consistently, previous studies also demonstrated the anti-obesity effect of SPX. Ge et al. [26] reported that SPX improves glucose tolerance and decreases insulin resistance and HbAlc in HFD-induced T2DM mice, and Sabrina and colleagues [27] also found that spexin decreases body weight and improves the metabolic profile and adipocyte hypertrophy. Collectively, all these studies indicated SPX may serve as a potentially useful agent to reduce obesity and improve glucose metabolism. However, the specific mechanism is not clear.

SPX has been highly implicated in appetite regulation and food consumption [17, 28]. In 2014, Jose et al. found that chronic subcutaneous injection with SPX reduced 32% of total caloric intake in diet-induced obesity rats [29]. Ha and colleagues also find the administration of SPX into the third ventricle near the hypothalamus significantly decreased food intake and body [30]. Interestingly, the food intake amount was comparable among each group throughout the study, which suggest the reason that SPX relieved body weight likely to increase energy expenditure rather than decrease energy intake.

As known, the main energy derivation pathways of the cell occur in mitochondria [31–33]. Our result displayed that SPX promoted the expression of mitochondrial biogenesis proteins (Cytochrome B) and the number of mitochondria in WAT of HFD-fed mice. Moreover, in vitro study also found after SPX treatment, the mature 3T3-L1 adipocytes transformed to a mitochondria-richer phenotype visualized from the result of mitochondrial fluorescent probe staining and transmission electron microscopic images. Therefore, these data indicate that the anti-obesity action of SPX is possibly mediated by increasing the energy expenditure of WAT.

As known, brown adipocytes possess numerous anatomical and molecular features different from white adipocytes. Brown or beige adipocytes have a multi-locular architecture with many small lipid droplets, more abundant mitochondria in the cytoplasm [34, 35], and distinct molecular markers, including TBX1 [36], CIDEA [37] and UCP-1 [38]. Notably, UCP-1 is a critical player in allowing electrons to be released rather than stored, resulting in heat release [39, 40]. White adipocytes have low UCP-1 content in basal conditions but when induced as browning have high UCP-1 expression levels and increased energy consumption [41]. Our data revealed that intraperitoneal treatment of SPX dramatically reduced adipocyte sizes, and up-regulated browning markers UCP-1, TBX1 and CIDEA in HFD-fed mice, suggesting a possible role of SPX in inducing the browning phenotype. Consistently, increased expression of adipocyte browning markers was observed in 3T3-L1 mature after SPX treatment. It is known the elevation of mitochondrial numbers is a critical characteristic feature of fat browning [31], and our data showed mitochondrial numbers in white adipocytes were promoted after SPX intervention, thus indicating SPX was a potent pro-browning inducer in white adipose. To our knowledge, this is the first study to reveal SPX ameliorated obesity partially by promoting the browning of white adipose.

The molecular mechanism underlying the browning effect of SPX is not clear yet. In this work, RNA-seq analysis displayed that the JAK/STAT signaling pathway was an important pathway involved in the browning action. It is known that JAK2 plays an important role in regulating UCP1 expression in brown adipose tissue [42], and the actions of JAK2-activating is mediated by STAT3 phosphorylation in BAT [43]. Indeed, the phosphorylation of JAK2 and STAT3 were upregulated by SPX both in HFD mice and in mature 3T3-L1 adipocytes. Similarly, Sally Yu Shi [44] also demonstrated that adipocyte JAK2-STAT3 is required for UCP1 induction in BAT and diet- and cold-induced thermogenesis. To further ascertain the mechanisms by which SPX promotes white adipose browning, we used JAK2 inhibitor AZD1480 and STAT3 inhibitor stattic to inhibit the JAK2-STAT3 pathway. Remarkably, AZD1480 or stattic abolished the pro-browning action of SPX on the upregulation of mitochondrial biogenesis and specific browning markers, including UCP-1, TBX1and CIDEA. These data indicate SPX promotes white adipose browning by activating the JAK2/STAT3 pathway. However, more exact mechanisms need to be further studied.

### Limitation

However, there are some limitations in the current study. Firstly, the food intake is the average food intake of mice in each cage. Since mice were not housed individually in the current study, the food intake of each animal cannot be recorded, which may cause bias in energy calculation. Secondly, although the optimal concentration of SPX used to treat adipocytes in vitro was selected according to previous literature and based on different stimulation concentrations, it should be mentioned the optimal concentration was supraphysiological, therefore, care should be taken in extrapolating in vitro results to in vivo studies.

## Conclusion

Taken together, we have for the first time demonstrated that SPX alleviated diet-induced obesity and ameliorated glucose and lipid metabolism by inducing the browning of white adipose, and the pro-browning action of SPX is mediated by JAK2/STAT3 pathway. These findings provide potential molecular targets for the treatment of obesity-related disorders. In addition, since brown adipocytes is a another main contributor to energy expenditure, whether SPX treatment could affect brown adipocytes still need further studied. Given the emerging advancement in the domain of small peptides, SPX may serve as a promising strategy for the treatment of obesity and metabolic diseases.

## Data Availability

Some datasets generated during and/or analyzed during the current study are not publicly available but are available from the corresponding author on reasonable request.
